# Predictive factors of restless leg syndrome in hemodialysis patients

**DOI:** 10.15171/jrip.2016.19

**Published:** 2016-05-20

**Authors:** Adel Eftekhari, Khadijeh Nasiriani, Samaneh Mirzaei, Somayeh Azimpour Ardakani

**Affiliations:** ^1^Department of Nursing, School of Nursing and Midwifery, Shahid Sadoughi University of Medical Sciences, Yazd, Iran; ^2^Department of Nursing, School of Nursing and Midwifery, Rafsanjan University of Medical Sciences, Rafsanjan, Iran

**Keywords:** Restless leg syndrome, Hemodialysis, Neurologic disorders

## Abstract

**Introduction:** The restless leg syndrome (RLS) is a neurologic disorder suffering the hemodialysis patients. Although the pathophysiology of this syndrome remains unknown yet, an investigation of the parameters pertinent to it may help to develop the related medical knowledge and to improve the therapeutic-care interventions in this regard.

**Objectives:** The correlation between the RLSs on individual, clinical, and laboratory indices in patients undergoing hemodialysis.

**Patients and Methods:** This descriptive-analytic study was conducted on 104 hemodialysis patients. Diagnosis of RLS was made using the International RLS Standard Questionnaire. The data on individual, clinical, and laboratory indices were obtained from patients’ recorded files and interviews.

**Results:** Based on our findings, 28.8% of the patients undergoing hemodialysis were affected with mild RLS, 41.7% with moderate RLS, and 29.5% with severe RLS. There was a statistically significant correlation between affliction with RLS on the one hand, and age and gender, on the other (*P* < 0.05). However, there was no significant correlation between RLS and education level, occupation, length of hemodialysis, fasting blood sugar (FBS), hepatitis B and C, serum blood urea nitrogen (BUN), creatinine (Cr), iron, hemoglobin (Hb) level and also KT/Vor URR (*P* < 0.05).

**Conclusion:** Regarding the high prevalence of RLS among the hemodialysis patients, there is the necessity for taking more care of these patients to reduce the somatic complications of the RLS especially among the elderly and female patients and to control the blood sugar of these patients at the normal level.

**Implication for health policy/practice/research/medical education:**

In the current study, we assessed the effects of clinical and laboratory indices and their changes in restless leg syndrome in hemodialysis patients. It is known that several laboratory indices affect this syndrome. The elevated level of blood glucose in these patients, measurement of the blood glucose index in these patients seems mandatory.

## Introduction

The restless leg syndrome (RLS) is a sensory-motor disturbance with increased inclination for moving the legs and sometimes other body organs accompanied by discomfort, distress, tingling, and numbness. It is aggravated with rest and lack of activity specifically during the night and relived with activity. Etiologically, it is divided into two types; type I or idiopathic RLS which usually follows a familial and genetic pattern, and type II which is associated with other disorders (1). One of these related disorders is renal failure and end-stage renal disease (nephropathy). The prevalence of RLS is estimated to be 6%-83% among the hemodialysis patients (2). The main cause of this syndrome is not known yet. It usually manifests itself in the dialysis patients during rest and disturbs and annoys the patient (3). The negative effects of this syndrome affecting life quality are reported as lack of comfort, sleeping disturbances, fatigue and exhaustion, and stress and anxiety which in turn, deteriorates the individual’s performance and influences the occupational and social activities and familial life, e.g., decreased capacity for environmental acclimatization or adaptation and problems in communicating with others (4). The important point here is the fact that this syndrome is common among the hemodialysis and renal failure patients. This motor impairment has not been considered satisfactorily (5,6). Considering the widely-accepted principle of “prevention is better than cure,” the recognition of influential factors in a disease process is rendered as the most primary steps in determining the treatment course. Due to reduced life quality, decreased life expectancy, and distorted body image in hemodialysis patients with restless leg syndrome, an awareness of the risk factors of their RLS is important. Nonetheless, a comprehensive search of electronic sources of the issue under consideration revealed that few influential studies have been conducted so far on factors related to RLS (5).

## Objectives

Regarding the rate of prevalence of RLS in hemodialysis patients, the present investigation was carried out to investigate the impacts of clinical and laboratory indices and their changes in these patients. It is hoped that some meticulous steps would be taken to improve the symptoms of RLS through an awareness of the related factors and ultimately promote the life quality of these patients.

## Patients and Methods

This descriptive-analytic study was conducted on 140 hemodialysis patients presenting to hospitals of Yazd, central Iran, during 2015. Having obtained informed written consent, the RLS was investigated in all hemodialysis patients via the International RLS Standard Questionnaire. This inventory consisted of 10 five-choice items each with a scale of 0-4 points (very severe, severe, moderate, mild, none). The severity of symptoms of this disorder were classified into five categories on the basis of acquired scores: without any problem (0 score), mild (1-10 score), moderate (11-20 score), severe (21-30 score), and very severe (31-40 score). Diagnosis of the RLS was established using the International RLS Standard Questionnaire. Further, the patients were examined by a nephrologist and a neurologist to exclude the differential diagnoses, so the included subjects suffered just from the RLS. Patients with a history of less than 3-month hemodialysis, a sensory-motor disorder, affliction with diabetic neuropathy, neurologic impairments, mental disorders, or affliction with cardiovascular diseases were excluded from the study. The data required for this section were gleaned with a three-section questionnaire. The first section of the questionnaire included demographic information such as age, gender, education, occupation, clinical information, and health history (i.e., cardiovascular diseases, diabetes, hypertension, hepatitis B, hepatitis C), length of hemodialysis, and analgesics consumption. Also, the second section included the laboratory indices. Seeing the regular performance of tests of hemodialysis patients by the hospital laboratories, the most recent clinical tests of these patients done in the intervals of hemodialysis, were considered in this study. The third section of the questionnaire was the standard screening instrument of the RLS introduced by the International Society of RLS Studies. Patients with the four criteria were rendered as RLS patients. The scientific credibility of the research instrument was established by the questionnaire reliability and validity estimation method presented by Habibzadeh et al and estimated to be 0.97 (5). Subsequently, the laboratory values including URR, KT/V, hemoglobin (Hb) level, Iron, serum creatinine (Cr), blood urea nitrogen (BUN), and fasting blood sugar (FBS) were compared to demographic information and clinical indices such as age, gender, education, occupation, health history and diseases, length of hemodialysis, and analgesics consumption. Then, they were analyzed for statistical correlation with RLS.

### Ethical issues

1) The research followed the tenets of the Declaration of Helsinki; 2) informed consent was obtained, and they were free to leave the study at any time and 3) the research was approved by the ethical committee of Shahid Sadoughi University of Medical Sciences and Health Services. To observe the ethical issues of research, the participating patients were informed of research process and objectives, and the questionnaires were filled out anonymously using interview by the researchers or by the use of patients’ records.

### Statistical analysis

Statistical analysis was performed using SPSS (version 16), statistical software pack­age. In this study, descriptive statistics were used to analyze the demographic profiles of the participants. A *P* value <0.05 was considered signif­icant. Chi-square test was used for comparison of categorical variables and analysis of variance (ANOVA) was used for the comparison of continuous variables.

## Results

A total of 139 patients including 54 females (38.8%) and 85 males (61.2%) were studied in this research. Forty patients suffered from mild RLS including 7 females (17.5%) and 33 males (82.5%), 58 patients had moderate RLS including 26 females (44.8%) and 32 males (55.2%), and 41 patients had severe RLS including 21 females (51.2%) and 20 males (48.8%). The prevalence of the syndrome in both genders was significantly different (*P*=0.004). Moreover, the mean ages of the patients with and without RLS were 60.99 and 55.47 years, respectively. The chi-square test revealed a statistically significant difference between age and prevalence of RLS (*P*=0.032), hence that RLS prevalence increased with increasing age (Table 1).

**Table 1:** Frequency of hemodialysis patients regarding affliction with RLS in terms of age and gender

**Number**	**Mild RLS**	**Moderate RLS**	**Severe RLS**	***P***
Gender				0.004
Male	7 (17.5%)	26 (44.8%)	21 (51.2%)	
Female	33 (82.5%)	32 (55.2%)	20 (48.8%)	
Age group				0.032
15-40	7 (17.5%)	7 (17.1%)	3 (5.2%)	
40-60	15 (37.5%)	8 (19.5%)	27 (46.6%)	
60+	18 (45.0%)	26 (63.4%)	28 (48.3%)	

Abbreviation: RLS, restless leg syndrome.

The length of hemodialysis period in patients with severe RLS and those with mild RLS was not significantly different (*P*=0.152). Yet, the hemodialysis time varied from 3-4 hours. Although the prevalence of RLS in patients with 3 hours dialysis was greater than those with 4 hours dialysis (35.7% versus 26.1%), the difference between the two groups was not significant(*P*>0.05). The results of ANOVA revealed that there was no significant difference between the patients with and without RLS with respect to BUN level, serum iron, Hb, serum Cr, FBS, and also KT/V, and URR (Table 2).

**Table 2:** Frequency of hemodialysis patients with RLS in terms of laboratory indices

**Variable**	**Mild RLS (n=40)**	**Moderate RLS (n=58)**	**Severe RLS (n=41)**	***P***
FBS (mg/dL)	124.35	146.90	158.24	0.077
BUN (mg/dL)	139.57	126.92	125.44	0.256
Cr (mg/dL)	8.092	7.158	7.410	0.193
Iron (ng/dL)	71.32	82.47	70.46	0.602
Hb (g/dL)	10.747	10.459	10.973	0.349
Kt/V	1.198	1.134	1.225	0.238
URR (%)	63.93	63.64	63.66	0.988

Abbreviations: RLS, restless leg syndrome; FBS; fasting blood sugar, BUN; blood urea nitrogen; Cr, creatinine; Hb, hemoglobin.

As seen, 12 patients (30%) with mild RLS, 26 patients (44.8%) with moderate RLS, and 17 patients (41.5%) with severe RLS used benzodiazepines, tricycle antidepressants, and GABA analogues. There was, however, no significant correlation between consumption of these drugs and the presence of the syndrome (*P*>0.05). Moreover, there were no significant correlations between a positive history of involvement by diabetes, hypertension, cardiovascular disease, hepatitis B, hepatitis C, and RLS (Table 3).

**Table 3:** Survey of history of diseases in hemodialysis patients regarding affliction with RLS

**Type of diseases**	**Mild RLS**	**Moderate RLS**	**Severe RLS**	***P ***
Diabetes	19 (47.5%)	32 (55.2%)	23 (56.1%)	0.687
Hypertension	22 (55%)	35 (60.3%)	25 (61.0%)	0.829
Cardiovascular	4 (10.0%)	7 (12.1%)	4 (9.8%)	0.919
Hepatitis B	2 (5.0%)	1 (1.7%)	1 (2.4%)	0.622
Hepatitis C	1 (2.4%)	2 (5.0%)	0 (0.0%)	0.244

Abbreviation: RLS, restless leg syndrome.

## Discussion

Compared to the general population, the RLS is more prevalent among patients with end-stage renal disease in both before and after dialysis phase (1). In our study, 71.2% of the hemodialysis patients were affected with RLS. The results of various studies have reported the prevalence of RLS differently among the RLS patients so that Samilipour et al reported the prevalence of this syndrome to be 33.1% among the dialysis patients (1). The study by Walker et al carried out in Canada in 1995 reported 33.3% prevalence of the syndrome among the hemodialytic patients (7). Additionally, the prevalence of RLS was 45.8% in England (8), 14.5% in Turkey (9), 14.8% in Brazil (10), 21.5% in Italy (11), 1.5%-6.6% in India (12,13), and 22.96% in Serbia (14).In the present study, there was a statistically significant difference in the prevalence of this syndrome between the two genders, the prevalence being greater among the males compared to females (*P*=0.004). This finding is consistent with the results of the studies conducted on hemodialytic patients in Canada (7), Torbat-e Heidarieh of Iran (4), and in Shahr-e Kord of Iran (15). This is, nevertheless, in contrast with the reported results of some studies reporting the prevalence of the syndrome to be greater among the female hemodialysis patients (16-21). Besides, some epidemiological studies indicate the tangibly higher prevalence of RLS among the female patients (22). Moreover, in most studies carried out on the general populations, increased prevalence of RLS has been associated with incasing age (16,23,24). This finding is consistent with our results, indicating a significant correlation between age and RLS prevalence. Yet, the studies by Siddiqui et al (8), Bhowmik et al (13), Alidosti et al (15) and Samilipour et al (1) did not report any significant correlation between age and prevalence of RLS. Our findings suggested further that there was no significant difference among the three groups of patients without mild RLS, without moderate RLS, and without severe RLS regarding education, occupation, history of disease (cardiovascular, diabetes, hypertension, hepatitis B, and hepatitis C), and consumption of analgesics. According to some studies, hypertension can function as a risk factor for RLS. The presence of various contradictory reports demands the completion of more future research for studying the role of hypertension or antihypertensive medications (25). Moreover, Alidosti et al found no significant difference among the three groups of patients with severe RLS, mild RLS, and patients without RLS with regard to age, sex, education, occupation, frequency of dialysis per week, and hemodialysis time. This finding is consistent with our findings (15). On the other hand, Telarović et al found that hemodialysis length is one of the risk factors of affliction with RLS (26). Yet, there was no significant difference between patients with and without RLS regarding length of dialysis in the studies by Samilipour et al (1) and Siddiqui et al (8). There was no significant difference among the three groups of patients with mild RLS, moderate RLS, and severe RLS regarding levels of FBS, BUN, Cr, iron, Hb, KT/V, and serum URR. Also, some other studies reported no correlation between BUN level and RLS (1,27,28). This is consistent with our results here. The studies on the association between serum iron and RLS reported varying results. A number of studies have confirmed the correlation between iron deficiency and RLS prevalence (29-31) while some other studies found no such a correlation. Continuous use of iron supplements in hemodialysis patients has obscured the correlation between iron and RLS among this group of patients (32,33). Furthermore, a significant correlation between Hb level and RLS in the studies by O’Keeffe et al was detected (34). Finally, the researcher found no study dealing with the standard correlation between KT/V, URR and RLS.

## Conclusion

The RLS is a common nuisance than a crippling disease among the chronic renal failure patients. Appropriate curative measures will promote the life quality of patients and improve their course of treatment. On The basis of the results of various studies, it is known that several laboratory indices affect this syndrome. Hence, it is logical to recommend the measurement and control of these indices. On the other hand, our findings demonstrated that the serum glucose level of patients with RLS is higher than that of patients without the syndrome though, of course, the difference was not significant. Considering the elevated level of blood glucose in these patients, measurement of the blood glucose index in these patients seems mandatory.

## Limitations of the study

The limitation of the current study was relatively small sample size.

## Acknowledgments

The author’s special thanks should go to the personnel and participating patients at Hemodialysis Ward of Shahid Rahnemoon Hospital in Yazd, Ziayee Hospital in Ardakan, and Imam Ja’far Sadiq Hospital in Meibod who sincerely cooperated with researchers during the completion of this research project.

## Authors’ contribution

AE; the concept, design, data analysis, and manuscript preparation. KHN; Study design, statistical analysis, manuscript editing, and manuscript review. SM and SAA; data collection.

## Conflicts of interest

The authors declared no competing interests.

## Ethical considerations

Ethical issues (including plagiarism, misconduct, data fabrication, falsification, double publication or submission, redundancy) have been completely observed by the authors.

## Funding/Support

This study was supported by the Deputy of Research of Shahid Sadoughi University of Medical Sciences (Grant # 3584).

## References

[1] Samilipour H, Azizi F, Motamed N, Taiebi N, Delavar Kasmaie H, Karimi M (2009). Restless leg syndrome and its association with serum ferritin in dialysis patients in Bushehr province, 2006. ISMJ.

[2] Hemmati Z, Alidosti M (2012). Frequency of restless legs syndrome in hemodialysis patients referring to Chaharmahal and Bakhtiari province hospitals. Journal of Health and Care.

[3] Winkelman JW, Chertow GM, Lazarus JM (2004). Restless legs syndrome in end-stage renal disease. Am J Kidney Dis.

[4] Shahidi AR, Abbaspour S, Namjou M, Najafzadeh A (2010). Restless legs syndrome in hemodialysis patients. JMCIRI.

[5] Lin CH, Wu VC, Li WY, Sy HN, Wu SL, Chang CC (2013). Restless legs syndrome in end‐stage renal disease: a multicenter study in Taiwan. Eur J Neurol.

[6] Giannaki CD, Sakkas GK, Hadjigeorgiou GM, Karatzaferi C, Patramani G, Lavdas E (2010). Non-pharmacological management of periodic limb movements during hemodialysis session in patients with uremic restless legs syndrome. ASAIO J.

[7] Walker S, Fine A, Kryger MH (1995). Sleep complaints are common in a dialysis unit. Am J Kidney Dis.

[8] Siddiqui S, Kavanagh D, Traynor J, Mak M, Deighan C, Geddes C (2005). Risk factors for restless legs syndrome in dialysis patients. Nephron Clin Pract.

[9] Soyoral Y, Sayarlioglu H, Tuncel D, Sahin M, Dogan E, Erkoc R (2010). Prevalence and risk factors of restless leg syndrome in a single hemodialysis unit. Turk J Med Sci.

[10] Goffredo Filho GS, Gorini CC, Purysko AS, Silva HC, Elias IE (2003). Restless legs syndrome in patients on chronic hemodialysis in a Brazilian city: frequency, biochemical findings and comorbidities. Arq Neuropsiquiatr.

[11] Gigli GL, Adorati M, Dolso P, Piani A, Valente M, Brotini S (2004). Restless legs syndrome in end-stage renal disease. Sleep Med.

[12] Bhowmik D, Bhatia M, Tiwari S, Mahajan S, Gupta S, Agarwal SK (2004). Low prevalence of restless legs syndrome in patients with advanced chronic renal failure in the Indian population: a case controlled study. Ren Fail.

[13] Bhowmik D, Bhatia M, Gupta S, Agarwal SK, Tiwari SC, Dash SC (2003). Restless legs syndrome in hemodialysis patients in India: a case controlled study. Sleep Med.

[14] Nikić PM, Andrić BR, Stojanović-Stanojević M, Dordević V, Petrović D, Stojimirović BB (2007). Restless legs syndrome prevalence in patients on chronic hemodialysis in central Serbia. Vojnosanit Pregl.

[15] Alidosti M, Hemate Z, Reisi M (2013). Relationship between the quality of sleep and restless legs syndrome among. KAUMS Journal (FEYZ).

[16] Lettieri CJ, Eliasson AH (2009). Pneumatic compression devices are an effective therapy for restless legs syndrome: a prospective, randomized, double-blinded, sham-controlled trial. Chest.

[17] Earley CJ (2003). Restless legs syndrome. N Engl J Med.

[18] Rothdach AJ, Trenkwalder C, Haberstock J, Keil U, Berger K (2000). Prevalence and risk factors of RLS in an elderly population The MEMO Study. Neurology.

[19] Berger K, Luedemann J, Trenkwalder C, John U, Kessler C (2004). Sex and the risk of restless legs syndrome in the general population. Arch Internal Med.

[20] Allen RP, Walters AS, Montplaisir J, Hening W, Myers A, Bell TJ (2005). Restless legs syndrome prevalence and impact: REST general population study. Arch Internal Med.

[21] Chavoshi F, Einollahi B, Haghighi KS, Saraei M, Izadianmehr N (201524). Prevalence and sleep related disorders of restless leg syndrome in hemodialysis patients. Nephrourol Mon.

[22] Fulda S, Stalla GK, Wetter TC (2007). Prevalence of the restless legs syndrome in transsexual patients: the hormonal hypothesis revisited. J Neurol.

[23] Rijsman R, Neven AK, Graffelman W, Kemp B, de Weerd A (2004). Epidemiology of restless legs in the Netherlands. Eur J Neurol.

[24] Nichols DA, Allen RP, Grauke JH, Brown JB, Rice ML, Hyde PR (2003). Restless legs syndrome symptoms in primary care: a prevalence study. Arch Internal Med.

[25] Batool-Anwar S, Malhotra A, Forman J, Winkelman J, Li Y, Gao X (2011). Restless legs syndrome and hypertension in middle-aged women. Hypertension.

[26] Telarović S, Relja M, Trkulja V (2007). Restless legs syndrome in hemodialysis patients: association with calcium antagonists. Eur Neurol.

[27] Collado-Seidel V, Kohnen R, Samtleben W, Hillebrand GF, Oertel WH, Trenkwalder C (1998). Clinical and biochemical findings in uremic patients with and without restless legs syndrome. Am J Kidney Dis.

[28] Winkelman JW, Chertow GM, Lazarus JM (1996). Restless legs syndrome in end-stage renal disease. Am J Kidney Dis.

[29] Sloand JA, Shelly MA, Feigin A, Bernstein P, Monk RD (2004). A double-blind, placebo-controlled trial of intravenous iron dextran therapy in patients with ESRD and restless legs syndrome. Am J Kidney Dis.

[30] Bastos JP, Sousa RB, Nepomuceno LA, Gutierrez-Adrianzen OA, Bruin PF, Araújo ML (2007). Sleep disturbances in patients on maintenance hemodialysis: role of dialysis shift. Revista da Associação Médica Brasileira.

[31] Habibzadeh H, Hemmati Maslakpak M, Ghanei Gheshlagh R (2012). The relationship between iron deficiency and restless legs syndrome in hemodialysis patients. J Shahid Sadoughi Univ Med Sci.

[32] Kim JM, Kwon HM, Lim CS, Kim YS, Lee SJ, Nam H (2008). Restless legs syndrome in patients on hemodialysis: symptom severity and risk factors. J Clin Neurol.

[33] Mucsi I, Molnar MZ, Ambrus C, Szeifert L, Kovacs AZ, Zoller R (2005). Restless legs syndrome, insomnia and quality of life in patients on maintenance dialysis. Dial Transplant.

[34] O’Keeffe ST, Gavin K, Lavan JN (1994). Iron status and restless legs syndrome in the elderly. Age Ageing.

